# Supplementary material for: Reliability and performance of the IRRAflow® system for intracranial lavage and evacuation of hematomas—A technical note
Source: PLoS One. 2024 Apr 16;19(4):e0297131. doi: 10.1371/journal.pone.0297131 (PMC11020765; doi:10.1371/journal.pone.0297131)
Supplement: S1 File — Figure c showing the bolted standard passive external ventricular drain (EVD), Speigelberg, Silverline®. (DOCX) [file pone.0297131.s001.docx]

**Supplementary material 1**. a and b showing the reverse tunneling and fastening of the IRRA*flow*® catheter. Figure c showing the bolted standard passive external ventricular drain (EVD), Speigelberg, Silverline®.

**
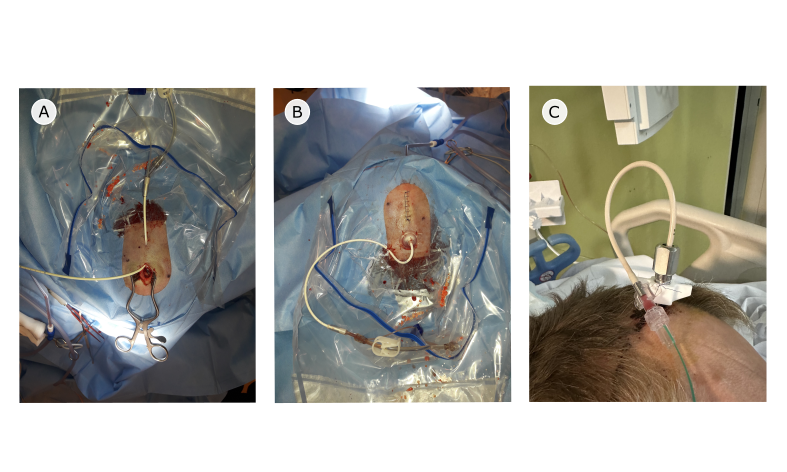
**
