# Supplementary material for: Reliability and performance of the IRRAflow® system for intracranial lavage and evacuation of hematomas—A technical note
Source: PLoS One. 2024 Apr 16;19(4):e0297131. doi: 10.1371/journal.pone.0297131 (PMC11020765; doi:10.1371/journal.pone.0297131)
Supplement: S2 File — (DOCX) [file pone.0297131.s002.docx]

**Supplementary material 2.** Room temperature, water amount, Device settings, and experiments repetitions in the Compliance test and Experiments 1-4.

| **Camber compliance test** |  |
| --- | --- |
| Room temperature (°celcius) | 23°celcius |
| Water in artificial brain (ml) | 100-600 ml (variable) |
| IRRAflow® lower alarm (mmhg) | Device not in use |
| IRRAflow® upper alarm (mmhg) | Device not in use |
| IRRAflow® drain above (mmhg) | Device not in use |
| IRRAflow® irrigation level (ml/hour) | Device not in use |
| IRRAflow® drainage bag level (cm) | Device not in use |
| Experiment repetitions | 5 times |
|  |  |
| **Experiment 1 – Bolt constriction test** |  |
| Room temperature (°celcius) | 23°celcius |
| Water in artificial brain (ml) | 200 ml |
| IRRAflow® lower alarm (mmhg) | - 99 mmhg |
| IRRAflow® upper alarm (mmhg) | + 99 mmhg |
| IRRAflow® drain above (mmhg) | - 99 mmhg |
| IRRAflow® irrigation level (ml/hour) | 180 ml/h |
| IRRAflow® drainage bag level (cm) | 29 cm |
| Duration of each experiment | 1 hour |
| Experiment repetitions | 5 times |
|  |  |
| **Experiment 2 – IRRAflow design space exploration** |  |
| Room temperature (°celcius) | 23°celcius |
| Water in artificial brain (ml) | 200 ml |
| IRRAflow® lower alarm (mmhg) | - 99 mmHg |
| IRRAflow® upper alarm (mmhg) | + 99 mmHg |
| IRRAflow® drain above (mmhg) | - 99 mmHg |
| IRRAflow® irrigation level (ml/hour) | Variable (20 ml/h, 90 ml/h and 180 ml/h) |
| IRRAflow® drainage bag level (cm) | Variable (+39 cm, 0 cm, -19, cm, -29 cm, -39 cm -49) |
| Duration of experiment at each setting | 10 minutes |
| Experiment repetitions | 5 times at each setting |
|  |  |
| **Experiment 3 - Accuracy of the IRRAflow injection volume** |  |
| Room temperature (°celcius) | 23°celcius |
| Water in artificial brain (ml) | 200 ml |
| IRRAflow® lower alarm (mmhg) | - 99 mmHg |
| IRRAflow® upper alarm (mmhg) | + 99 mmHg |
| IRRAflow® drain above (mmhg) | - 99 mmHg |
| IRRAflow® bolus setting | 1 ml |
|  |  |
| **Experiment 4 - Accuracy and reliability of the IRRAflow pressure sensor** |  |
| Room temperature (°celcius) | 23°celcius |
| Water in artificial brain (ml) | 200 ml |
| IRRAflow® lower alarm (mmhg) | - 99 mmHg |
| IRRAflow® upper alarm (mmhg) | + 99 mmHg |
| IRRAflow® drain above (mmhg) | - 99 mmHg |
| IRRAflow® bolus setting | 1 ml |
