# Supplementary material for: Reliability and performance of the IRRAflow® system for intracranial lavage and evacuation of hematomas—A technical note
Source: PLoS One. 2024 Apr 16;19(4):e0297131. doi: 10.1371/journal.pone.0297131 (PMC11020765; doi:10.1371/journal.pone.0297131)
Supplement: S3 File — (SysS: System status, TrtS: treatment status, ICP: intracranial pressure, Cprs: current pressure, HLim: Upper alarm limit, LLim: Lower alarm limit, PM: Preset Mode (1 or 0), (system operating on default settings or not, PPrs: Preset pressure (drain above), Ivol: infusion volume (ml/hour), CycT: cycle time (sec), stat1 HEX printout of various system status, Stat2: HEX printout of various system status, ErrCode: Errorcode). (DOCX) [file pone.0297131.s003.docx]

**Supplementary material 3.** Data output from the IRRAflow control unit. (SysS: System status , TrtS: treatment status, ICP: intracranial pressure, Cprs: current pressure, HLim: Upper alarm limit, LLim: Lower alarm limit, PM: Preset Mode (1 or 0), (system operating on default settings or not, PPrs: Preset pressure (drain above), Ivol: infusion volume (ml/hour), CycT: cycle time (sec), stat1 HEX printout of various system status, Stat2: HEX printout of various system status, ErrCode: Errorcode)

| **Date** | **Time** | **SysS** | **TrtS** | **Alarm** | **ICP** | **Cprs** | **HLim** | **LLim** | **PM** | **PPrs** | **Ivol** | **CycT** | **Stat1** | **Stat2** | **ErrCode** |
| --- | --- | --- | --- | --- | --- | --- | --- | --- | --- | --- | --- | --- | --- | --- | --- |
| 01.05.2022 | 00:05:58 | Trt | Meas | None | 3,9 | 3,2 | 46 | -20 | 0 | 10 | 0 | 40 | 0x1E50 | 0x0C60 | 0 |
| 01.05.2022 | 00:06:03 | Trt | Meas | None | 3 | 3 | 46 | -20 | 0 | 10 | 0 | 40 | 0x1E50 | 0x0C60 | 0 |
| 01.05.2022 | 00:06:08 | Trt | Meas | None | 3 | 3,4 | 46 | -20 | 0 | 10 | 0 | 40 | 0x1E50 | 0x0C60 | 0 |
| 01.05.2022 | 00:06:13 | Trt | Meas | None | 3 | 3,9 | 46 | -20 | 0 | 10 | 0 | 40 | 0x1E50 | 0x0C60 | 0 |
| 01.05.2022 | 00:06:18 | Trt | Meas | None | 3 | 4,2 | 46 | -20 | 0 | 10 | 0 | 40 | 0x1E50 | 0x0C60 | 0 |
| 01.05.2022 | 00:06:24 | Trt | Meas | None | 3,8 | 4,4 | 46 | -20 | 0 | 10 | 0 | 40 | 0x1E50 | 0x0C60 | 0 |
| 01.05.2022 | 00:06:29 | Trt | Meas | None | 3,8 | 4,3 | 46 | -20 | 0 | 10 | 0 | 40 | 0x1E50 | 0x0C60 | 0 |
| 01.05.2022 | 00:06:34 | Trt | Meas | None | 3,8 | 3,7 | 46 | -20 | 0 | 10 | 0 | 40 | 0x1E50 | 0x0C60 | 0 |
| 01.05.2022 | 00:06:39 | Trt | Meas | None | 3,4 | 4,3 | 46 | -20 | 0 | 10 | 0 | 40 | 0x1E50 | 0x0C60 | 0 |
| 01.05.2022 | 00:06:44 | Trt | Meas | None | 3,4 | 4,4 | 46 | -20 | 0 | 10 | 0 | 40 | 0x1E50 | 0x0C60 | 0 |
| 01.05.2022 | 00:06:49 | Trt | Meas | None | 4,2 | 4,2 | 46 | -20 | 0 | 10 | 0 | 40 | 0x1E50 | 0x0C60 | 0 |
| 01.05.2022 | 00:06:54 | Trt | Meas | None | 4,2 | 4,1 | 46 | -20 | 0 | 10 | 0 | 40 | 0x1E50 | 0x0C60 | 0 |
| 01.05.2022 | 00:06:59 | Trt | Meas | None | 4,1 | 4,5 | 46 | -20 | 0 | 10 | 0 | 40 | 0x1E50 | 0x0C60 | 0 |
| 01.05.2022 | 00:07:04 | Trt | Meas | None | 4,3 | 4,2 | 46 | -20 | 0 | 10 | 0 | 40 | 0x1E50 | 0x0C60 | 0 |
| 01.05.2022 | 00:07:09 | Trt | Meas | None | 4,3 | 4,9 | 46 | -20 | 0 | 10 | 0 | 40 | 0x1E50 | 0x0C60 | 0 |
| 01.05.2022 | 00:07:14 | Trt | Meas | None | 4,9 | 5,2 | 46 | -20 | 0 | 10 | 0 | 40 | 0x1E50 | 0x0C60 | 0 |
| 01.05.2022 | 00:07:19 | Trt | Meas | None | 4,9 | 4,8 | 46 | -20 | 0 | 10 | 0 | 40 | 0x1E50 | 0x0C60 | 0 |
| 01.05.2022 | 00:07:24 | Trt | Meas | None | 4 | 4,5 | 46 | -20 | 0 | 10 | 0 | 40 | 0x1E50 | 0x0C60 | 0 |
| 01.05.2022 | 00:07:29 | Trt | Meas | None | 4 | 4,3 | 46 | -20 | 0 | 10 | 0 | 40 | 0x1E50 | 0x0C60 | 0 |
| 01.05.2022 | 00:07:34 | Trt | Meas | None | 4,6 | 4,2 | 46 | -20 | 0 | 10 | 0 | 40 | 0x1E50 | 0x0C60 | 0 |
| 01.05.2022 | 00:07:39 | Trt | Meas | None | 4,6 | 4,3 | 46 | -20 | 0 | 10 | 0 | 40 | 0x1E50 | 0x0C60 | 0 |
| 01.05.2022 | 00:07:44 | Trt | Meas | None | 4 | 4,1 | 46 | -20 | 0 | 10 | 0 | 40 | 0x1E50 | 0x0C60 | 0 |
| 01.05.2022 | 00:07:49 | Trt | Meas | None | 5 | 4,6 | 46 | -20 | 0 | 10 | 0 | 40 | 0x1E50 | 0x0C60 | 0 |
| 01.05.2022 | 00:07:54 | Trt | Meas | None | 5 | 5,8 | 46 | -20 | 0 | 10 | 0 | 40 | 0x1E50 | 0x0C60 | 0 |
| 01.05.2022 | 00:07:59 | Trt | Meas | None | 5,5 | 5,1 | 46 | -20 | 0 | 10 | 0 | 40 | 0x1E50 | 0x0C60 | 0 |
| 01.05.2022 | 00:08:05 | Trt | Meas | None | 5,5 | 9,3 | 46 | -20 | 0 | 10 | 0 | 40 | 0x1E50 | 0x0C60 | 0 |
| 01.05.2022 | 00:08:10 | Trt | Meas | None | 3,7 | 3,7 | 46 | -20 | 0 | 10 | 0 | 40 | 0x1E50 | 0x0C60 | 0 |
| 01.05.2022 | 00:08:15 | Trt | Meas | None | 3,7 | 3,3 | 46 | -20 | 0 | 10 | 0 | 40 | 0x1E50 | 0x0C60 | 0 |
| 01.05.2022 | 00:08:20 | Trt | Meas | None | 3 | 2,8 | 46 | -20 | 0 | 10 | 0 | 40 | 0x1E50 | 0x0C60 | 0 |
| 01.05.2022 | 00:08:25 | Trt | Meas | None | 3 | 3 | 46 | -20 | 0 | 10 | 0 | 40 | 0x1E50 | 0x0C60 | 0 |
