# Supplementary figures and images for: Reliability and performance of the IRRAflow® system for intracranial lavage and evacuation of hematomas—A technical note
Source: PLoS One. 2024 Apr 16;19(4):e0297131. doi: 10.1371/journal.pone.0297131 (PMC11020765; doi:10.1371/journal.pone.0297131)

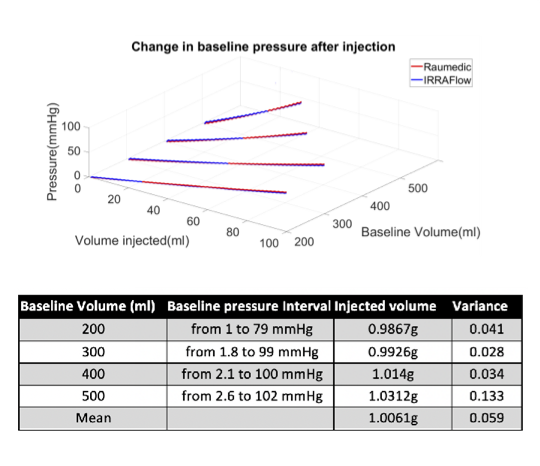

Supplement: S1 Fig — (TIFF) [file pone.0297131.s005.tiff]
